# RK4 and HAM Solutions of Eyring–Powell Fluid Coating Material with Temperature-Dependent-Viscosity Impact of Porous Matrix on Wire Coating Filled in Coating Die: Cylindrical Co-ordinates

**DOI:** 10.3390/polym13213696

**Published:** 2021-10-27

**Authors:** Zeeshan Khan, Waris Khan, Ilyas Khan, Nawa Alshammari, Nawaf N. Hamadneh

**Affiliations:** 1 Department of Mathematics and Statistics, Bacha Khan University, Charsadda 24420, Pakistan; 2 Department of Mathematics, Hazara University, Mansehra 25000, Pakistan; wariskhan758@yahoo.com; 3 Department of Mathematics, College of Science, Al-Zulfi, Majmaah University, Majmaah 11952, Saudi Arabia; 4 Department of Basic Sciences, College of Science and Theoretical Studies, Saudi Electronic University, Riyadh 11673, Saudi Arabia; n.alshammari@seu.edu.sa (N.A.); nhamadneh@seu.edu.sa (N.N.H.)

**Keywords:** RK4 and HAM solutions, Eyring–Powell fluid, non-Newtonian fluid, transverse MHD effect, permeable matrix, temperature-dependent viscosity

## Abstract

In this work, we studied the impacts of transmitting light, nonlinear thermal, and micropolar fluid mechanics on a wire surface coating utilizing non-Newtonian viscoelastic flow. Models with temperature-dependent variable viscosity were used. The boundary layer equations governing the flow and heat transport processes were solved using the Runge–Kutta fourth order method. A distinguished constituent of this study was the use of a porous matrix that acted as an insulator to reduce heat loss. In this paper we discuss the effects of numerous development parameters, including $\beta_{0}$, Q, m, Ω, Kp, and Br (non-Newtonian parameter, heat-producing parameter, viscosity parameter, variable viscosity parameter, porosity parameter, and Brinkman number, respectively). Furthermore, the effects of two other parameters, *D* and *M*, are also discussed as they relate to velocity and temperature distributions. We observed that the velocity profiles decreased with increasing values of Kp. Fluid velocity increased as the values of M, Br, N, and D increased, while it decreased when the values of Kp, Q and D increased. For increasing values of M, the temperature profile showed increasing behavior, while Br and Q showed decreasing behavior. Furthermore, the present work is validated by comparison with HAM and previously published work, with good results.

## 1. Introduction

**Figure 1:**
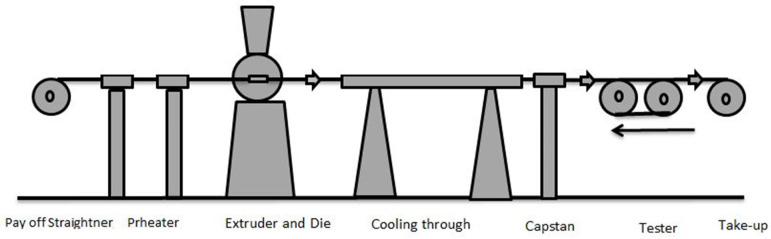
A typical wire-coating process.

Many of the fluids that engineers and scientists interact with are Newtonian fluids (e.g., air, water, oil). However, in many circumstances, the foundation of Newtonian behavior is non-rational and complex, necessitating the development of non-Newtonian responses. Non-Newtonian fluid behavior can be found in a variety of fluid materials, such as glue, custard, paint, blood, and ketchup. Many studies have emphasized the importance of non-Newtonian fluids because of their wide variety of engineering and industrial applications [1,2,3,4,5,6,7,8]. Rahman et al. [9] investigated non-Newtonian nanofluids in arterial supply through compounded stenosis. Eyring–Powell fluid, first proposed in 1944 by Eyring and Powell, is one such fluid. Several characteristics of Eyring–Powell fluids have been studied [10,11,12,13,14]. The wire-coating procedure is critical for preventing injuries and reducing the damage caused by machine vibration. Various melt polymers are used to coat wire in industries. In most cases, two techniques are needed to coat wire. In the first step, melt polymer is continuously dropped on the wire, and in the second step the wire is dragged through a die soaked with viscoelastic material. The coaxial process, dripping method, and electrostatic deposition process are the three processes utilized for wire coating. The dipping phase in the wire-coating process creates a considerably stronger link between the continua, but it is slower than the other two operations. Figure 1 depicts the geometry of the wire-coating procedure. A payoff device, straightener, heating element, extrusion device and die, cooling device, capstan, tester, and pull reel are all included. The bare wire is wrapped on the payoff device that goes through the straightener, while the wire is heated via a preheater, and a nozzle die contains a classical die where the molten polymer is assembled and coated. After that, the coating wire is chilled by a cooling unit, then passed through a capstan and a tester before being wound on a take-up reel. Various non-Newtonian fluids have been used by numerous investigators to examine coating processes [15,16,17,18,19,20,21,22,23].

Magnetohydrodynamics deals with highly conductive flow characteristics in the applied magnetic field. Many scholars have dedicated a significant amount of time to the study of magnetohydrodynamic flow problems [24,25,26,27,28,29,30]. Due to its Lorentz force, an applied magnetic field produces a current that has a significant impact on fluid motion. The fluid moment is reduced due to this Lorentz force. Magnetohydrodynamics has recently gained prominence as a research issue due to its widespread application in a variety of industrial processes, such as magnetic field material processing and glassmaking.

Researchers are interested in fluid flow through porous media because of the wide range of engineering applications. Porous media include carbonated pebbles, wood, metal foams, and other well-known materials. They have many industrial and residential uses, including as filters, printing papers, fuel cells, and batteries, which now use a very thin porous layer. Porous media have received a great deal of attention in several studies [31,32,33].

The heat exchange of non-Newtonian flow fields has gained popularity due to its potential applications in a wide range of industries. Rehman et al. [34] investigated heat exchange research for 3D stagnation point flow. The effect of heat exchange investigation and magnetohydrodynamic fluid was explored by many other scholars [35,36,37,38,39,40,41,42,43]. Hamid et al. [44] studied the heat transfer of a nanofluid through a permeable plate with radiation along slip conditions. Similarly, Hamid et al. [45,46] investigated the heat transfer of pseudoplastic through a permeable surface in the presence of nanoparticles. Tanveer et al. [47] studied the chemical reactions and heat-transfer rate of a micropolar fluid passing over a convectively heated sheet. Muhammad et al. [48] studied a Casson nanofluid over a stretching sheet. Some recent research about heat transfer can be seen in [49,50].

Nobody has yet examined the coating process for wire using a magnetohydrodynamic Eyring–Powell fluid. The goal of this work was to use Reynolds’ and Vogel’s models to describe the wire-coating process as it relates to heat production, porous materials, and variable viscosity.

## 2. Wire Coating Modelling

**Figure 2:**
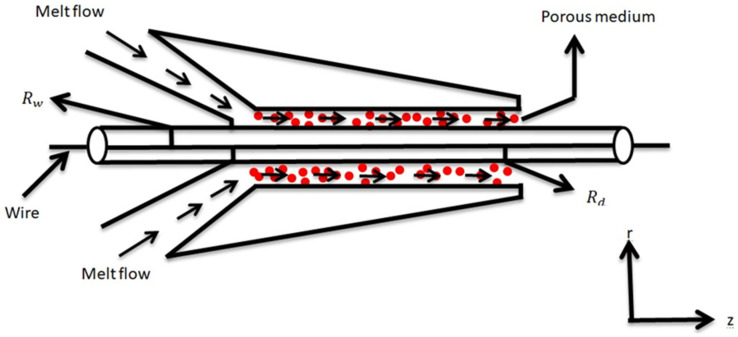
Geometry of the problem.

Figure 2 depicts the geometry of the problem under investigation. Here, L denotes the length of the die, $R_{d}$ denotes the radius, and $\theta_{d}$ denotes the temperature where an inviscid polymer melt is saturated. Where the temperature of the wire is determined by $\theta_{w}$, radius $R_{d}$, and velocity $U_{w}$ in porous medium, the wire is dragged at the center line a fixed pressurized die. A constant pressure gradient dp analogous to the x-axis and a normal magnetic field of strength $B_{0}$ work together to operate the emerging fluid. The induced magnetic number is used as a minor in our current scenario in order to ignore the actual magnetization. The reference problem is formulated along the wire axis.

The proper formulas for fluid velocity, stress tensor, and temperature field in the above-mentioned circumstances are as follows [17,18,19,20]:

(1) $$q=0\underline{i}+0j+w\left(r\right)k$$

(2) S=S(r)

(3) θ=θ(r)

For viscoelastic Eyring–Powell fluid, the Cauchy stress tensor is written as [17,18,19,20]:

(4) $$S=\mu *\nabla v+\frac{1}{\beta }{*\sinh}^{-1}\left(\frac{1}{C}\nabla v\right)$$

Equation (4) can be simplified as follows:

(5) $$\sinh^{-1}\left(\frac{1}{C}*\nabla v\right)\approx \frac{1}{C}*\nabla v-\frac{1}{6}{\left(\frac{1}{C}*\nabla v\right)}^{3},\;\left|\frac{1}{C}*\nabla v\right|\ll 1$$

For the sake of this discussion, the appropriate boundary conditions are as follows [17,20]:

(6) $$w\left(R_{w}\right)=U_{w},\;\theta \left(R_{w}\right)=\theta_{w}$$

The fundamental equations follow as [17,18,19,20]:

(7) ∇⋅q=0

(8) $$\rho \left(\frac{Dq}{Dt}\right)=F-\nabla p+J*B+\frac{\mu q}{K_{p}^{*}}$$

(9) $$\rho C_{p}\frac{D\theta }{Dt}=k\nabla^{2}+\varphi +Q_{0}\left(\theta -\theta_{w}\right)+Jd$$

where ***q,*** Dt/D, J∗B, ρ, and $Q_{0}$ are the velocity vector, material derivative, magnetic field, density, and rate of volumetric heat generation, respectively.

The magnetic body force generated in the z-direction is defined as [18,20]:

(10) $$J*B=\left(0,0,\sigma \beta_{0}^{2}w\right)$$

Equation (7) is satisfied automatically when (1–3) are used, and non-zero terms are as follows:

(11) $$S_{zr}=\left(\mu +\frac{1}{\beta C}\right)\frac{dw}{\;dr}-\frac{1}{6\beta C^{3}}{\left(\frac{dw}{\;dr}\right)}^{3}$$

(12) $$\frac{\partial P}{\partial r}=0$$

(13) $$\frac{\partial P}{\partial \theta }=0$$

(14) $$\frac{\partial P}{\partial z}=\frac{1}{r}\frac{d}{dr}\left[r\left\{\left(\mu +\frac{1}{\beta C}\right)\frac{dw}{\;dr}-\frac{1}{6\beta C}{\left(\frac{dw}{\;dr}\right)}^{3}\right\}\right]-\sigma \beta_{0}^{2}w-\frac{\mu w}{K_{p}^{*}}$$

However, due to the pressure gradient, Equation (14) displays the flow. When departing the die, the only event occurring is wire drag. As a result, pressure gradient has no effect and Equation (14) becomes:

(15) $$\frac{1}{r}\frac{d}{dr}\left[r\left\{\left(\mu +\frac{1}{\beta C}\right)\frac{dw}{\;dr}-\frac{1}{6\beta C}{\left(\frac{dw}{\;dr}\right)}^{3}\right\}\right]-\sigma \beta_{0}^{2}w-\frac{\mu w}{K_{p}^{*}}=0$$

Energy Equation (9) becomes:

(16) $$\begin{array}{l} K\left(\frac{d^{2}\theta }{dr^{2}}+\frac{1}{r}\frac{d\theta }{dr}\right)\\ +\left(\left(\mu +\frac{1}{\beta C}\right)\frac{dw}{\;dr}-\frac{1}{6\beta C^{3}}{\left(\frac{dw}{\;dr}\right)}^{3}\right),\\ \frac{dw}{\;dr}+Q_{0}\left(\theta -\theta_{w}\right)+\sigma \beta_{0}^{2}w^{2}=0 \end{array}$$

## 3. Constant Viscosity

Dimensionless parameters are defined as [17,18,19,20]:

(17) $$\begin{array}{l} r^{*}=\frac{r}{R_{w}},\;w^{*}=\frac{w}{U_{w}},\;M^{2}=\frac{\sigma \beta_{0}^{2}R_{w}^{2}}{\mu },\\ K_{p}=\frac{R_{w}^{2}}{K_{p}^{*}},\;w=\frac{v_{0}}{U_{w}},\;N=\frac{1}{\mu \beta C},\\ \theta_{w}^{*}=\frac{\left(\theta -\theta_{w}\right)}{\left(\theta_{d}-\theta_{w}\right)},\;Q=\frac{Q_{0}R_{w}^{2}}{K},\\ B_{r}=\frac{\mu U_{w}^{2}}{K\left(\theta_{d}-\theta_{w}\right)},\;R_{w}=\frac{\beta v_{0}}{\mu },\;\varepsilon =\frac{\mu }{6w^{2}{\left(\beta C\right)}^{3}} \end{array}$$

We derive the following form by introducing the above new variables in Equations (6), (15) and (16):

(18) $$\left(1+N\right)\left[r\frac{d^{2}w}{\;dr^{2}}+\frac{dw}{\;dr}\right]-\varepsilon \left[{\left(\frac{dw}{\;dr}\right)}^{3}+3r{\left(\frac{dw}{\;dr}\right)}^{2}\frac{d^{2}w}{\;dr^{2}}\right]-M^{2}wr-K_{p}wr=0$$

(19) w(1)=1 and w(δ)=0

(20) $$\frac{d^{2}\theta }{dr^{2}}+\frac{1}{r}\frac{d\theta }{dr}+B_{r}\left(1+N\right){\left(\frac{dw}{\;dr}\right)}^{2}+\varepsilon B_{r}{\left(\frac{dw}{\;dr}\right)}^{4}+Q\theta +B_{r}M^{2}w^{2}=0$$

(21) θ(1)=0 and θ(δ)=1

## 4. Temperature-Dependent Viscosity

Reynolds’ model is utilized to explain temperature-dependent viscosity in this case. For Reynolds’ model, viscosity in dimensionless form is [17]:

(22) $$\mu \approx 1-\beta_{0}m\theta$$

It is used to calculate temperature-dependent viscosity variation, with m as the viscosity parameter. We introduce the following non-dimensional parameters [17,18,19,20]:

(23) $$\begin{array}{c} \eta^{*}=\frac{r}{R_{w}},\;f^{*}=\frac{w}{U_{w}},\;M^{2}=\frac{\sigma \beta_{0}^{2}R_{w}^{2}}{\mu_{0}},K_{p}=\frac{R_{w}^{2}}{K_{p}^{*}},\;f=\frac{v_{0}}{U_{w}},\;N=\frac{1}{\mu_{0}\beta C},\;\mu^{*}=\frac{\mu }{\mu_{0}},\theta^{*}=\\ \frac{\left(\theta -\theta_{w}\right)}{\left(\theta_{d}-\theta_{w}\right)},\;Q=\frac{Q_{0}R_{w}^{2}}{K},B_{r}=\frac{\mu_{0}U_{w}^{2}}{K\left(\theta_{d}-\theta_{w}\right)},\;R_{f}=\frac{\beta v_{0}}{\mu_{0}},\;\varepsilon =\frac{\mu_{0}}{6f^{2}{\left(\beta C\right)}^{3}} \end{array}$$

In view of Equation (23), Equations (18)–(21), after deleing the asterisks, become:

(24) $$\begin{array}{c} \frac{d^{2}f}{d\eta^{2}}\left[\eta \left(1-\beta_{0}m\theta \right)+\eta N-3\eta \varepsilon {\left(\frac{df}{d\eta }\right)}^{2}\right]+\frac{df}{d\eta }\left[1-\beta_{0}m\theta +N-\beta_{0}m\eta \frac{d\theta }{d\eta }\right]\\ -\varepsilon {\left(\frac{df}{d\eta }\right)}^{3}-K_{p}f\eta -M^{2}f\eta =0 \end{array}$$

(25) f(1)=1 and f(δ)=0

(26) $$\frac{d^{2}\theta }{d\eta^{2}}+\frac{1}{\eta }\frac{d\theta }{d\eta }+\left(1-\beta_{0}m\theta \right)B_{r}{\left(\frac{df}{\;d\eta }\right)}^{2}+B_{r}{\left(\frac{df}{\;d\eta }\right)}^{2}\left(N+\varepsilon \right)+Q\theta +B_{r}M^{2}f^{2}=0$$

(27) θ(1)=0 and θ(δ)=1

For Vogel’s model [17]:

(28) $$\mu =\mu_{0}\exp\left(\frac{D}{B^{\prime }+\theta }-\theta_{f}\right)$$

After expanding Equation (28), we have:

(29) $$\mu =\Omega \left(1-\frac{D}{B^{\prime 2}}\theta \right)$$

Here, *D* and *B* are viscosity parameters and $\Omega =\mu_{0}\exp\left(\frac{D}{B^{\prime 2}}-\theta_{f}\right).$ After deleting the asterisks, Equations (24)–(27) become:

(30) $$\begin{array}{cc} & \frac{d^{2}f}{\;d\eta^{2}}\left[\eta \Omega \left(1-\frac{D}{B^{\prime 2}}\theta \right)+\eta N-3\eta \varepsilon {\left(\frac{df}{\;d\eta }\right)}^{2}\right]\\ & +\frac{df}{\;d\eta }\left[\Omega \left(1-\frac{D}{B^{2}}\theta \right)+N-\Omega \frac{D}{B^{\prime 2}}\eta \frac{d\theta }{d\eta }\right]\\ & -\varepsilon {\left(\frac{df}{\;d\eta }\right)}^{3}-K_{p}f\eta -M^{2}f\eta =0 \end{array}$$

(31) f(1)=1,f(δ)=0

(32) $$\begin{array}{cc} & \frac{d^{2}\theta }{d\eta^{2}}+\frac{1}{\eta }\frac{d\theta }{dr}+\Omega \left(1-\frac{D}{B^{\prime 2}}\theta \right)B_{r}{\left(\frac{df}{\;d\eta }\right)}^{2}\\ & +B_{r}{\left(\frac{df}{\;d\eta }\right)}^{2}\left(N+\varepsilon \right)+Q\theta +B_{r}M^{2}f^{2}=0 \end{array}$$

(33) θ(1)=0 and θ(δ)=1

## 5. Numerical Computation

### Constant Viscosity

Equations (30)–(33), which regulate the system, are reduced to first ODE.

The RK4 approach is used to solve them numerically [17,18]:

(34) $$\frac{d^{2}f}{\;d\eta^{2}}=\frac{\varepsilon {\left(\frac{df}{\;d\eta }\right)}^{3}-\left(1+N\right)\frac{df}{\;d\eta }+M^{2}f\eta +K_{p}f\eta }{\left(1+N\right)\eta +3\eta \varepsilon {\left(\frac{df}{\;d\eta }\right)}^{2}}$$

(35) $$\frac{d^{2}\theta }{d\eta^{2}}=-\left[\frac{1}{\eta }\frac{d\theta }{d\eta }+B_{r}\left(1+N\right){\left(\frac{df}{\;d\eta }\right)}^{2}+\varepsilon B_{r}{\left(\frac{df}{\;d\eta }\right)}^{4}+Q\theta +B_{r}M^{2}f^{2}\right]$$

We introduce the following new variables:

(36) $$f=y_{1},\;f^{\prime }=y_{2},\;f^{\prime \prime }=y_{2}^{\prime },\;\theta =y_{3},\;\theta^{\prime }=y_{4},\;\theta^{\prime \prime }=y_{4}^{\prime }$$

(37) $$y_{2}^{\prime }=\frac{\varepsilon {\left(y_{2}\right)}^{3}-\left(1+N\right)y_{2}+M^{2}y_{1}\eta +K_{p}y_{1}\eta }{\left(1+N\right)\eta +3\eta \varepsilon {\left(y_{2}\right)}^{2}}$$

(38) $$y_{4}^{\prime }=-\left[\frac{1}{\eta }y_{4}+B_{r}\left(1+N\right){\left(y_{2}\right)}^{2}+\varepsilon B_{r}{\left(y_{2}\right)}^{4}+Qy_{3}+B_{r}M^{2}y_{1}^{2}\right]$$

The boundary conditions become:

(39) $$y_{1}\left(1\right)=1\;\mathrm{and}\;y_{1}\left(\delta \right)=0$$

(40) $$y_{3}\left(1\right)=0\;\mathrm{and}\;y_{3}\left(\delta \right)=1$$

Similarly, for Reynolds’ and Vogel’s models, we transform the higher ODE into a first-order ODE as:

For Reynolds’ model:

(41) $$\begin{array}{ll} y_{2}^{\prime }= & \frac{\varepsilon {\left(y_{2}\right)}^{3}+K_{p}y_{1}r+M^{2}y_{1}r-y_{2}\left[1-\beta_{0}my_{3}+N-\beta_{0}mry_{4}\right]}{\left[r\left(1-\beta_{0}my_{3}\right)+rN-3r\varepsilon {\left(y_{2}\right)}^{2}\right]},\\ y_{4}^{\prime }= & -[\frac{1}{r}y_{4}+\left(1-\beta_{0}my_{3}\right)B_{r}{\left(y_{2}\right)}^{2}\\ & +B_{r}{\left(y_{2}\right)}^{2}\left(N+\varepsilon \right)+Qy_{3}+B_{r}M^{2}y_{1}^{2}], \end{array}$$

(42) $$y_{1}\left(1\right)=1\;\mathrm{and}\;y_{1}\left(\delta \right)=0$$

(43) $$y_{3}\left(1\right)=0\;\mathrm{and}\;y_{3}\left(\delta \right)=1$$

For Vogel’s model:

(44) $$\begin{array}{ll} y_{2}^{\prime }= & \frac{\varepsilon {\left(y_{2}\right)}^{3}+K_{p}y_{1}r+M^{2}y_{1}r-y_{2}\left[\Omega \left(1-\frac{D}{B^{2}}y_{3}\right)+N-\Omega \frac{D}{B^{2}}ry_{4}\right]}{r\Omega \left(1-\frac{D}{B^{2}}y_{3}\right)+rN-3r\varepsilon {\left(y_{2}\right)}^{2}}\\ y_{4}^{\prime }= & -[\frac{1}{r}y_{4}+\Omega \left(1-\frac{D}{B^{\prime 2}}y_{3}\right)B_{r}{\left(y_{2}\right)}^{2}\\ & +B_{r}{\left(y_{2}\right)}^{2}\left(N+\varepsilon \right)+Qy_{3}+B_{r}M^{2}y_{1}^{2}] \end{array}$$

(45) $$y_{1}\left(1\right)=1\;\mathrm{and}\;y_{1}\left(\delta \right)=0$$

(46) $$y_{3}\left(1\right)=0\;\mathrm{and}\;y_{3}\left(\delta \right)=1$$

## 6. Results and Discussion

**Figure 3:**
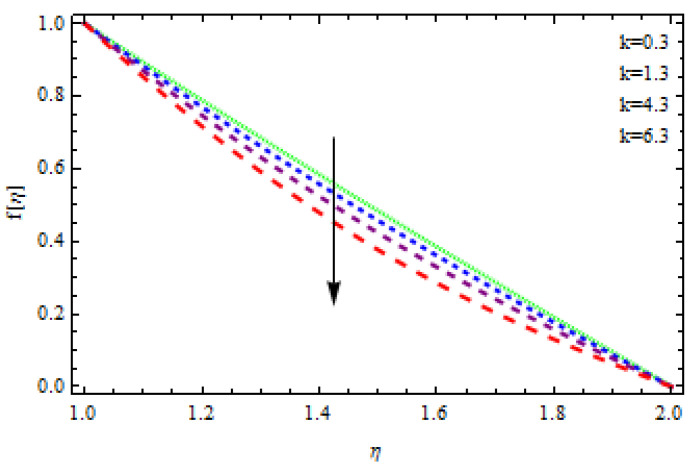
Influence of *k* on the velocity curve.

**Figure 4:**
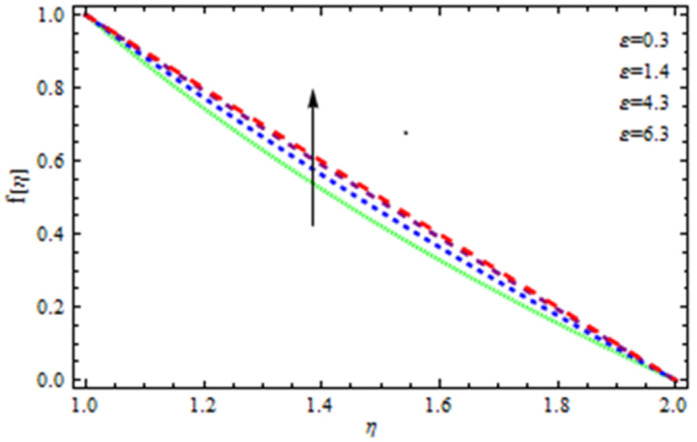
Influence of *ε* on the velocity curve.

**Figure 5:**
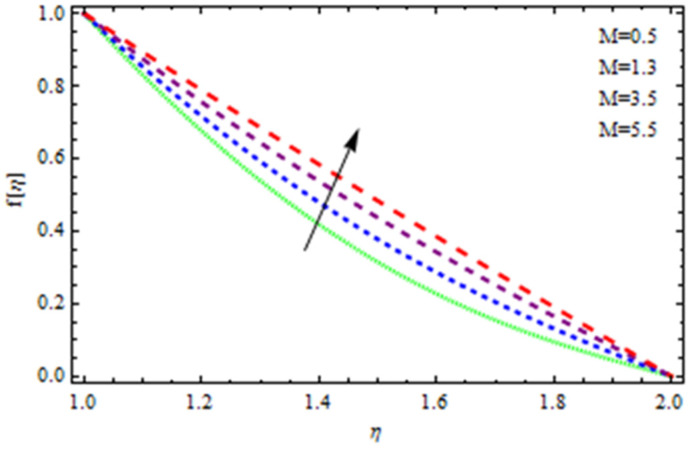
Influence of *M* on the velocity curve.

**Figure 6:**
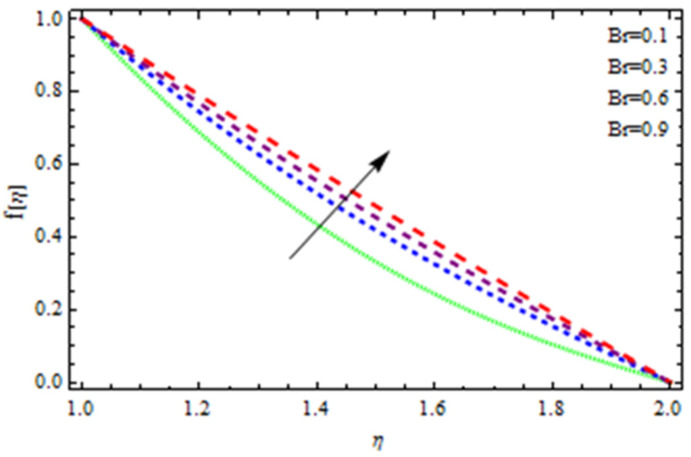
Influence of *Br* on the velocity curve.

**Figure 7:**
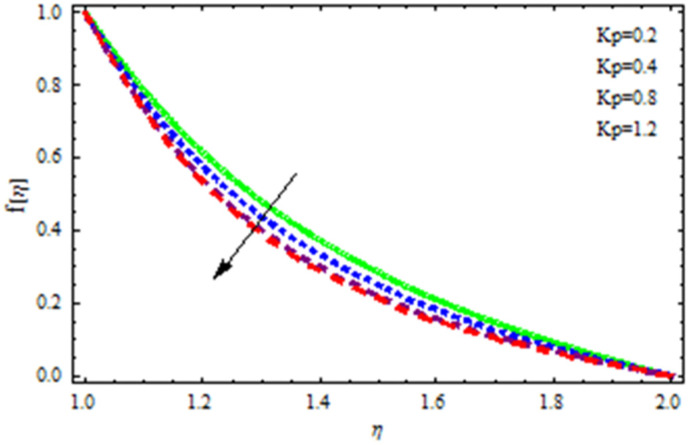
Influence of *Kp* on the velocity curve in the case of the Reynolds model.

**Figure 8:**
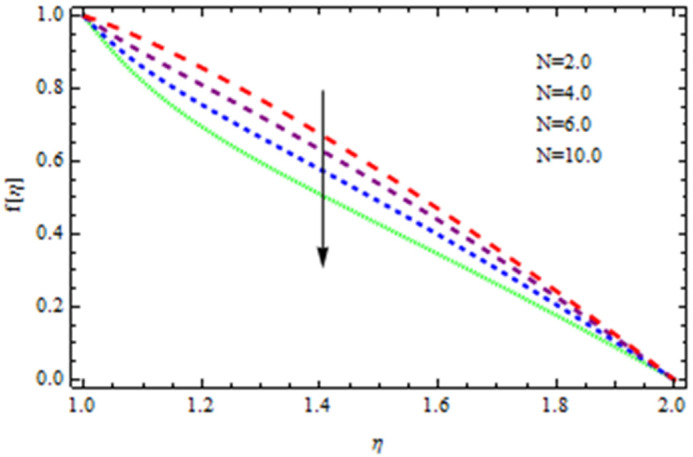
Influence of *N* on the velocity curve in the case of the Reynolds model.

**Figure 9:**
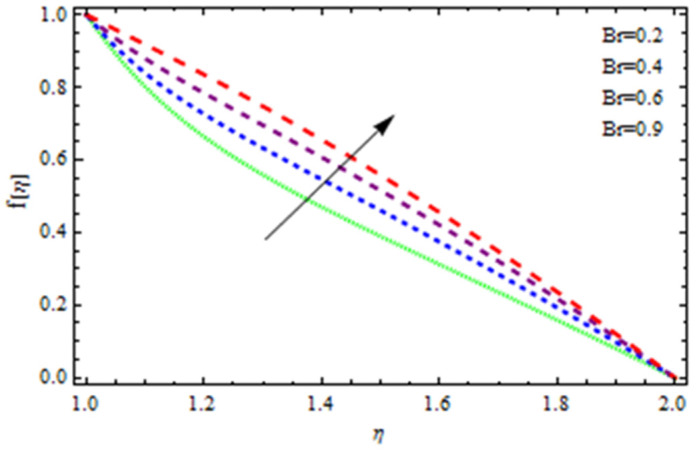
Influence of *Br* on the velocity curve in the case of Vogel’s model.

**Figure 10:**
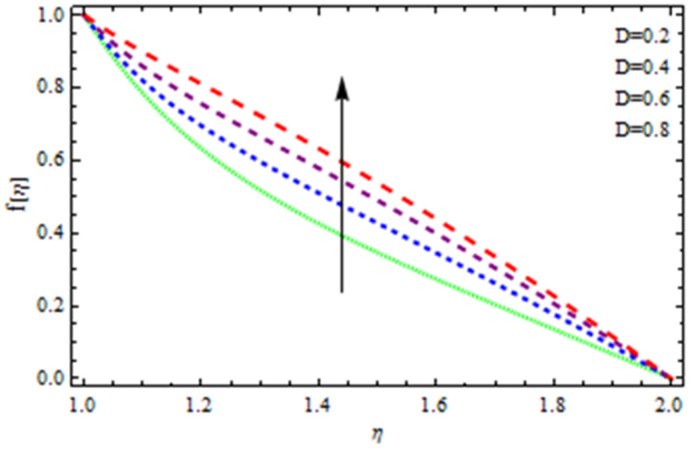
Influence of *D* on the velocity curve in the case of Vogel’s model.

**Figure 11:**
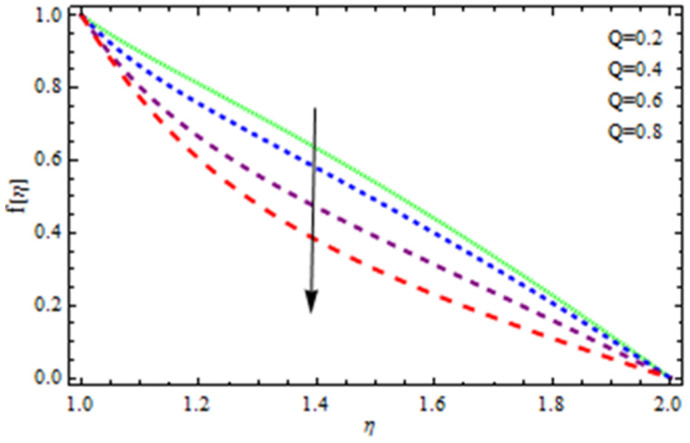
Influence of *Q* on the velocity curve in the case of Vogel’s model.

**Figure 12:**
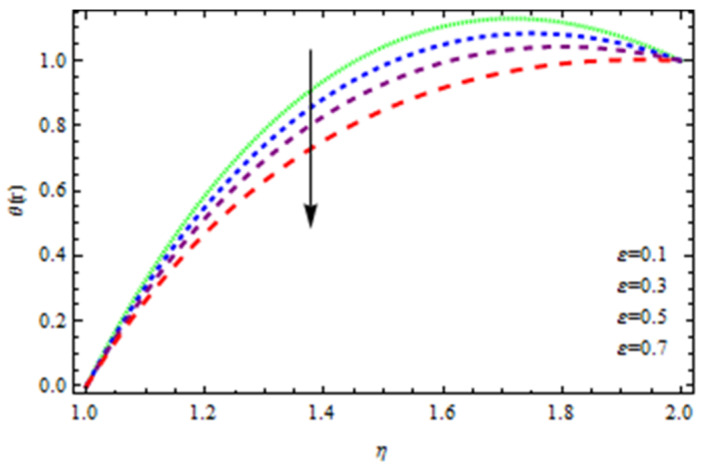
Influence of *ϵ* on the temperature curve.

**Figure 13:**
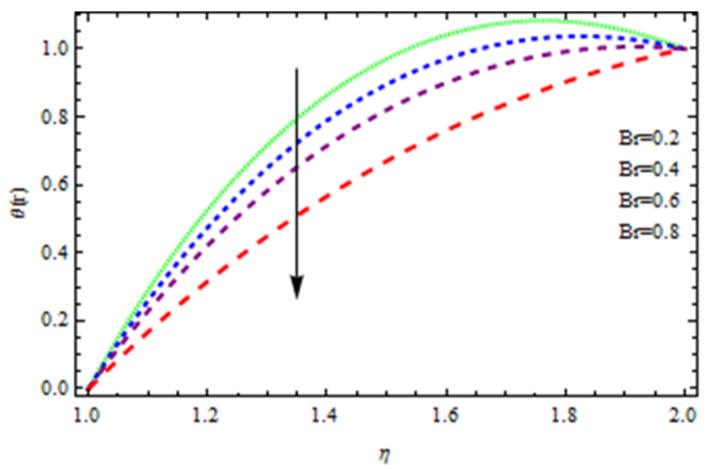
Influence of *Br* on the temperature curve.

**Figure 14:**
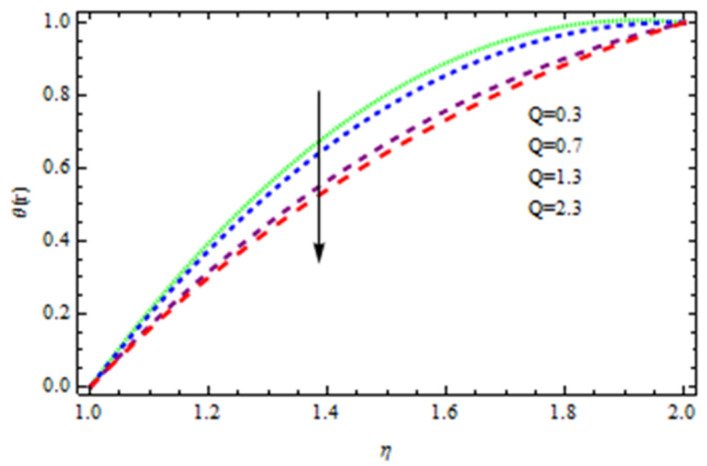
Influence of *Q* on the temperature curve.

**Figure 15:**
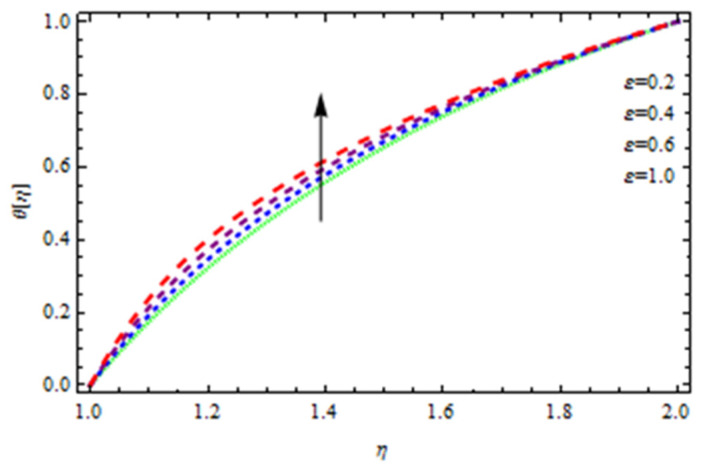
Temperature distribution in the Reynolds model for *ϵ*.

**Figure 16:**
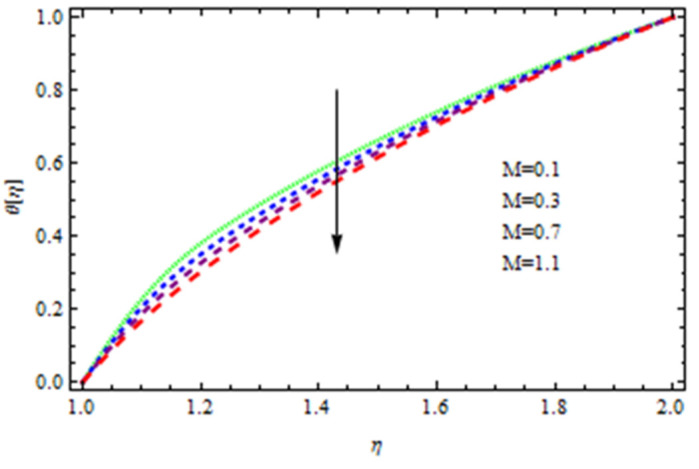
Temperature distribution in the Reynolds model for *M*.

**Figure 17:**
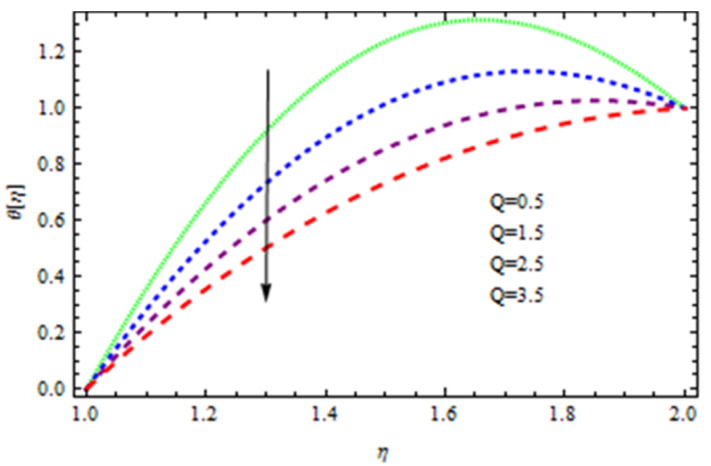
Temperature distribution in the Reynolds model for *Q*.

**Figure 18:**
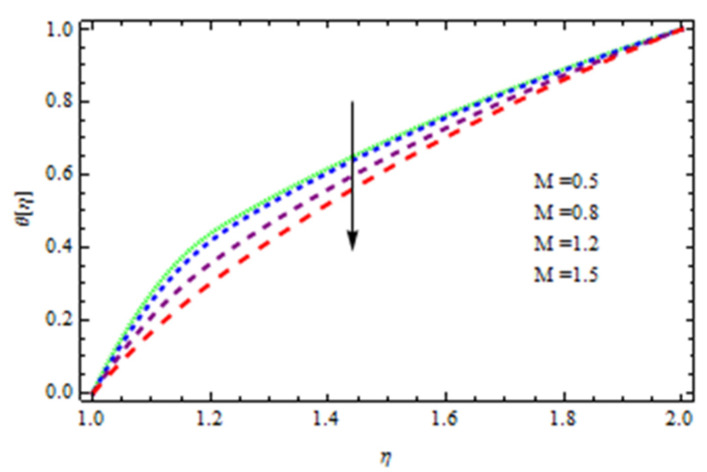
Temperature distribution in Vogel’s model for *M*.

**Figure 19:**
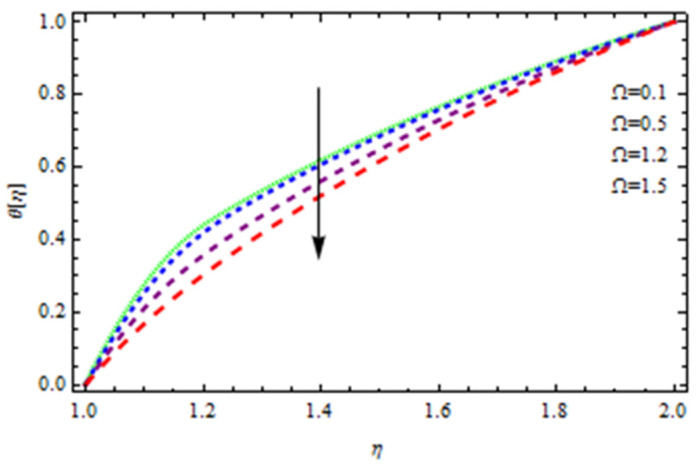
Temperature distribution in Vogel’s model for *Ω*.

**Figure 20:**
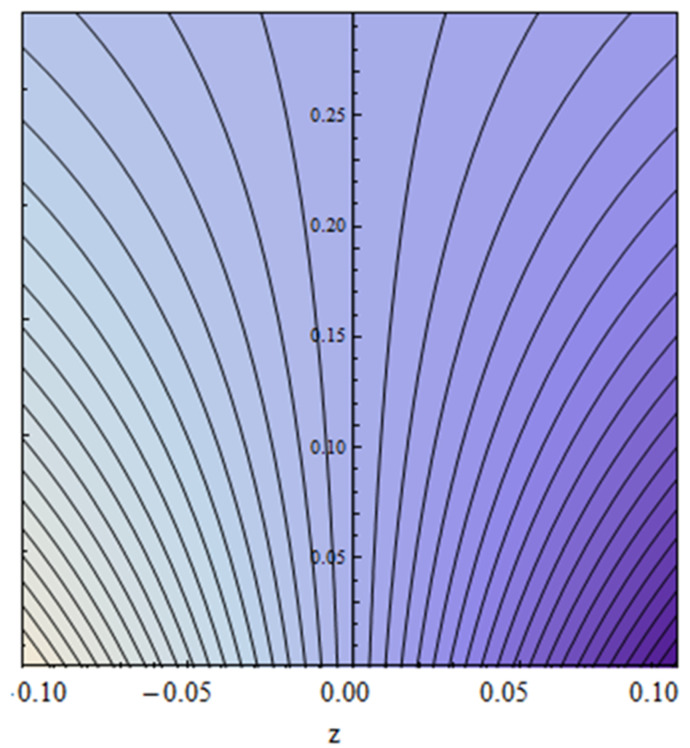
Streamlines impact at *Br* = 0.3 for constant viscosity.

**Figure 21:**
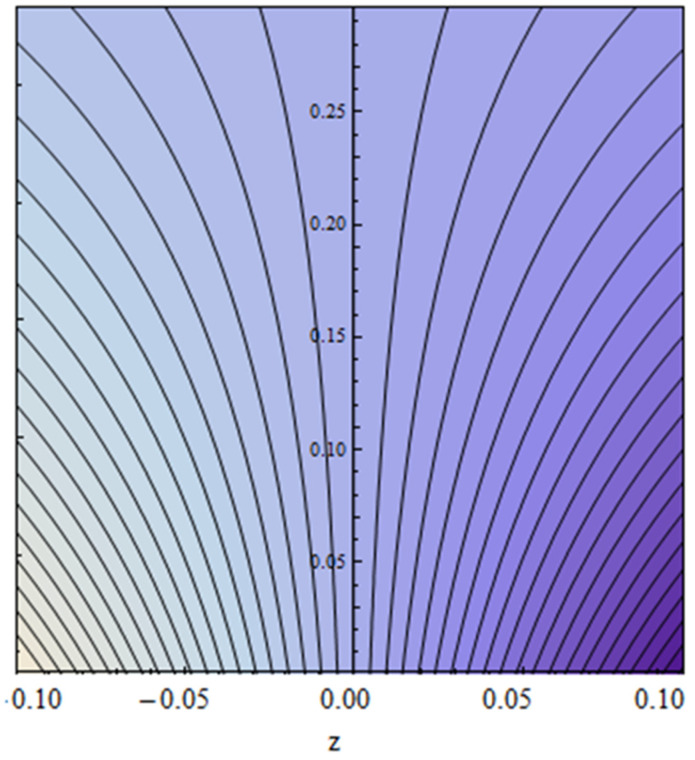
Streamlines impact at *Br* = 0.5 for the Reynolds model.

**Figure 22:**
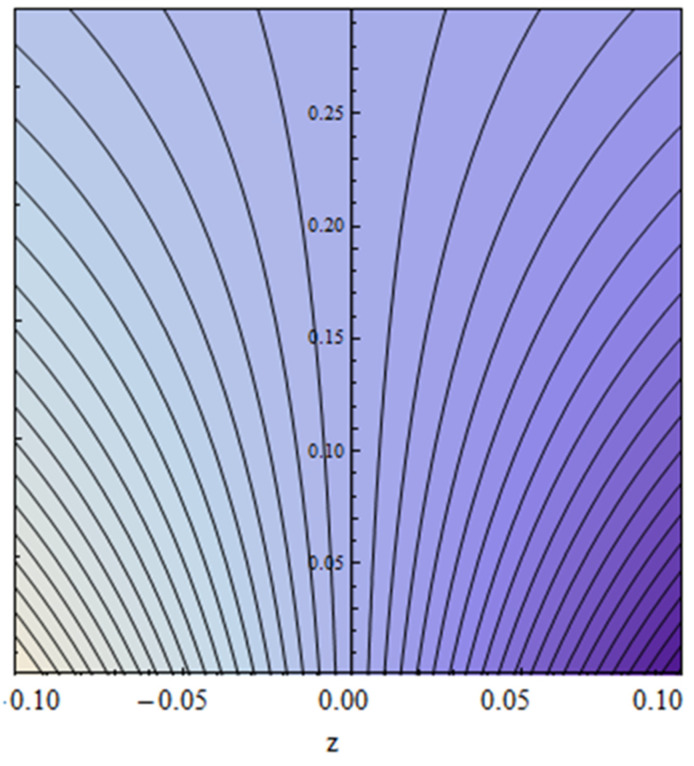
Streamlines impact at *Br* = 0.6 for Vogel’s model.

An EP (Eyring–Powell) fluid was examined for wire surface coating in this study. In a porous medium, wire coating usually happens inside a die with persistent magnetic and heat emission effects. We examined the effects of numerous development parameters, including Q, m, Ω, Kp, and Br, which stand for the non-Newtonian parameter, heat-producing parameter, viscosity parameter, variable-viscosity parameter, porosity parameter, and the Brinkman number, respectively. Furthermore, the effect of two other parameters, D and M, are also discussed as they relate to velocity and temperature distributions. Keeping Br and  Q fixed, Figure 3 shows the effect of Kp on velocity distribution in the absence of variable viscosity. The velocity significantly decreased with increasing Kp. Ignoring variable viscosity and keeping other parameters fixed, Figure 4 shows the effect of increasing *ɛ* on the velocity distribution. With increasing *ɛ*, the velocity profile shows an increasing tendency. The influence of M on velocity distribution is displayed in Figure 5. In the figure, the velocity profile increases as the parameter *M* increases. The impact of Br on the velocity distribution in the Reynolds model is shown in Figure 6. The Brinkman number (*Br*) is a dimensionless parameter used during melt processing to describe the conduction of heat from a barrier to a moving viscous fluid. With increasing Br, the velocity profile increased as well. This is attributed to the increased thermal power produced by viscous dissipation, which increases the fluid temperature and, as a result, increases the buoyant force. As a consequence, increasing the buoyant force causes the velocity to increase as well. When *β*_0_ = 0.2, *M* = 0.6, *Br* = 0.1, *Q* = 0.1, and *m* = 0.3, Figure 7 depicts the effects of the permeability parameter on the velocity in the case of the Reynolds model. As *Kp* increased, the velocity inside the die decreased. It is self-evident that the existence of a porous material generates greater fluid dynamic restriction, causing the flow to decline. As a result, as the impermeability factor increases, the barrier to flow velocity increases, and therefore velocity falls. Figure 8 shows the effect of *N* on the velocity profile for the Reynolds model. The growing action caused by a rise in *N* is eliminated by the velocity curve. Figure 9 illustrates that in Vogel’s model, the velocity of the fluid exhibits an increase with increasing *Br*, with *M* = 0.1, *Kp* = 0.6, and *Q* = 0.6. In Vogel’s model, the velocity curve in Figure 10 depicts an increasing tendency with increasing *D*. Figure 11 shows the tendency in the velocity distribution as *Q* increases in the case of Vogel’s model, with *D* = 0.3, *M* = 0.1, and *Br* = 0.5. When *M* = 0.5, *Kp* = 0.6, and *N* = 0.02 in the case of constant viscosity, Figure 12 illustrates the fluctuations in temperature distribution caused by *ɛ*. With increasing *ɛ*, the velocity profile shifted downward. The influence of the Brinkman number (*Br*) on the temperature curve with constant viscosity is shown in Figure 13; the dimensionless temperature profile falls as the dimensionless factor *Br* is increased in Figure 13, implying that this parameter enhances the wall temperature more than the mean temperature. This is because the fluid flow transports relatively less energy nearer to the borders in comparison to the core area, which may be the result of greater temperatures near the wall area. With viscosity and all other parameters held constant, Figure 14 shows the impact of *Q* on the temperature profile. With increasing *Q*, the fluid velocity shows an increasing tendency. Figure 15 shows an increasing behavior of the temperature field as the value of *ɛ* increases, in the case of the Reynolds model. In Figure 16, as *M* increases, the temperature profile increases as well. For the Reynolds model, the temperature profiles show an increasing behavior as *M* increases. By applying a perpendicular magnetic field to an electrical conductor fluid, a Lorentz force is created. The resulting Lorentz force has the capacity to eliminate fluid velocity in a confined geometry while also causing a temperature increase. As a result, increasing the value of the magnetic field parameter causes the thickness of the thermal boundary layer to develop, but the speed in the flow direction drops. With increasing *Q*, the temperature curve shows a decreasing behavior in Figure 17. In the case of Vogel’s model with *D* = 0.5, *Kp* = 0.2, and *Q* = 0.6, Figure 18 shows that the temperature inside the die increased due to impedance in *M*. Figure 19 illustrates that increasing *Ω* led to a declining temperature trajectory in Vogel’s model with *N* = 0.3, *β*_0_ = 1.2, *Kp* = 0.2, and *D* = 0.5. The influence on the streamlines of various values of *Br* with uniform velocity is shown in Figure 20. Figure 21 shows the impact on streamlines of different values of *Br* in the case of the Reynolds model. Figure 22 shows how streamlines are affected by the variation of *Br* in Vogel’s model.

## 7. Validation of the Results

**Figure 23:**
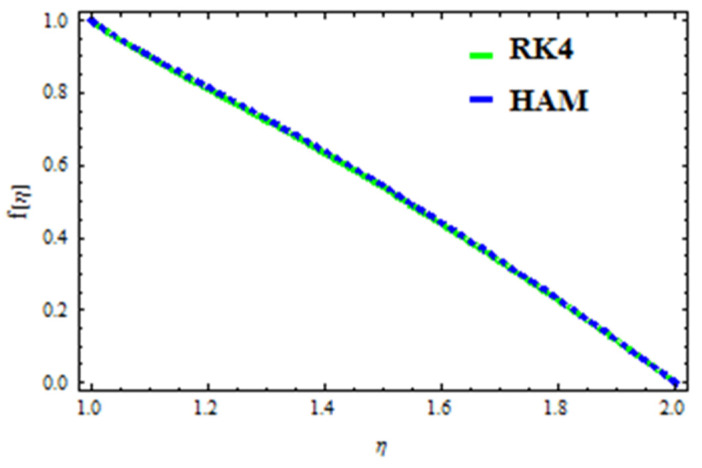
Comparison of RK4 and HAM for velocity under constant viscosity.

**Figure 24:**
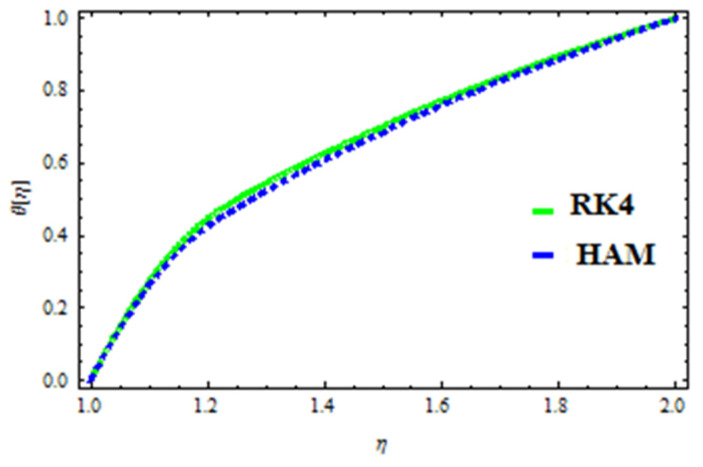
Comparison of RK4 and HAM for temperature under constant viscosity.

**Figure 25:**
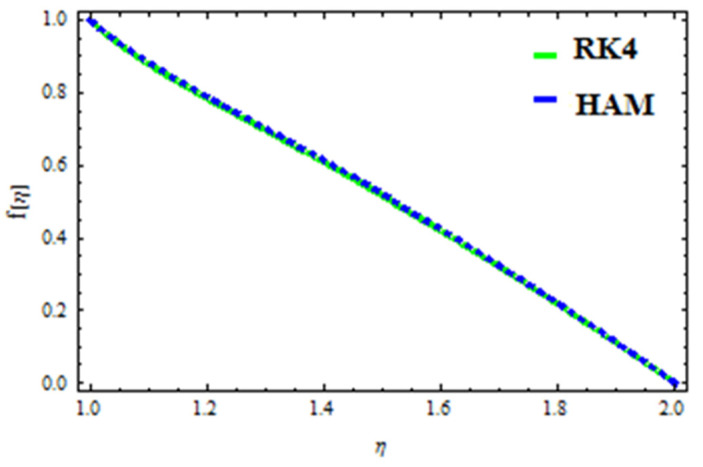
Comparison of RK4 and HAM for velocity in Reynolds’ model.

**Figure 26:**
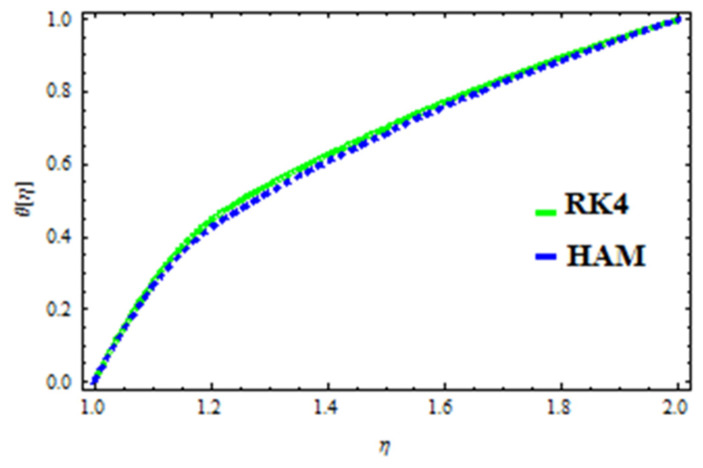
Comparison of RK4 and HAM for temperature in Reynolds’ model.

**Figure 27:**
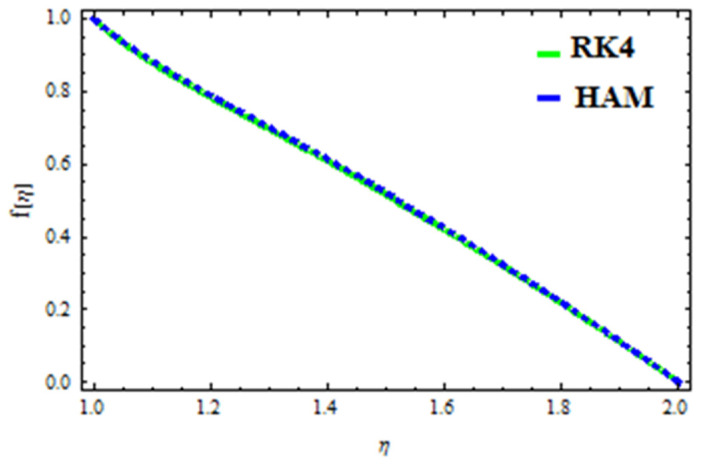
Comparison of RK4 and HAM for velocity in Vogel’s model.

**Figure 28:**
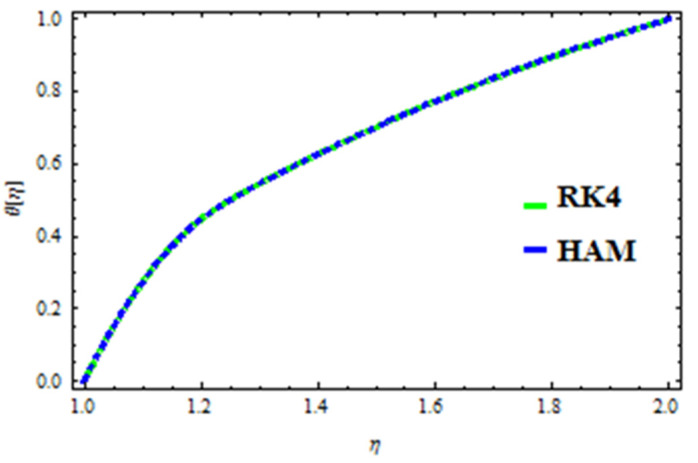
Comparison of RK4 and HAM for temperature in Vogel’s model.

A HAM approach was used for confirmation of the results. As demonstrated in Figure 23, Figure 24, Figure 25, Figure 26, Figure 27 and Figure 28, we had excellent agreement using this method. This comparative study was carried out for three different scenarios: constant viscosity, Vogel’s model, and Reynolds’ model. In addition, as shown in Table 1, the residual was calculated. The current results were also validated with the published work of Hayat et al. [11] to ensure the accuracy of our findings. This analysis revealed that our results obtained for the stated model parameters were in remarkable conformity, and we are sure of the veracity and flexibility of our conclusions.

## 8. Conclusions

We calculated the influence of an MHD fluid as well as heat transmission on the coating of wire utilizing a viscoelastic fluid in the presence of a porous medium. The impact of temperature-dependent viscosity and Joule heating were also discussed. The wire was coated with an Eyring–Powell fluid in a pressure-type die. Because a porous matrix was employed as an insulator, the heat and mass flexibility process reduced heat loss, thus improving the melting capacity. The numerical approach was utilized to obtain a numerical solution to the given model. Regarding velocity and temperature profiles, the outcomes of the involved parameters were shown. Increases in the values of *ɛ*, *M*, *Br*, *N*, and *D* caused the fluid velocity to increase, whereas increases in the values of *Kp*, *Q*, and *D* caused fluid velocity to decrease. For the Reynolds model, the temperature profiles depicted increasing behavior as *M* increased. By applying a perpendicular magnetic field to an electrical conductor fluid, the Lorentz force is created. The resulting Lorentz force has the capacity to eliminate fluid velocity in a confined geometry while also causing a rise in temperature. As a result, raising the magnetic field parameter causes the thickness of the thermal boundary layer to develop, but the speed in the flow direction to drop. When increasing *Br*, the temperature profiles decreased. This is because the fluid flow transports relatively less energy nearer to the borders in comparison to the core area, which may be the result of greater temperatures near the wall area. The temperature profile indicated an increasing tendency in the values of *ɛ* and *M*, as well as a declining behavior in the values of *Br* and *Q*. In addition, the present study was also validated with HAM and compared with published literature, and the results were in good agreement.

## Figures and Tables

**Table 1:** Comparison of the present work with HAM and published works.

η	RK4	HAM	Hayat et al. [11]	Absolute Error
**0.0**	1	1	1	0
**0.1**	0.906702	0.906701	0.906702	1.7263$\times 10^{-5}$
**0.2**	0.798963	0.798962	0.798963	3.1826$\times 10^{-6}$
**0.3**	0.676887	0.676885	0.676887	5.2213$\times 10^{-10}$
**0.4**	0.543737	0.543736	0.543737	1.7120$\times 10^{-11}$
**0.5**	0.406571	0.406571	0.406571	0.00327$\times 10^{-21}$
**0.6**	0.275849	0.275849	0.275849	0.10240$\times 10^{-21}$
**0.7**	0.163688	0.163689	0.163688	0.25100$\times 10^{-22}$
**0.8**	0.080481	0.080481	0.0804805	1.0021$\times 10^{-30}$
**0.9**	0.0296124	0.0296124	0.0296124	1.00010$\times 10^{-33}$
**1.0**	5.34328 × 10^−12^	5.34328 × 10^−12^	5.34328 × 10^−12^	0.00152$\times 10^{-33}$

## Data Availability

There is no data to support this research.
